# Synthesis and Thermoelectric Properties of Selenium Nanoparticles Coated with PEDOT:PSS

**DOI:** 10.3390/polym11061052

**Published:** 2019-06-17

**Authors:** Chingu Kim, Jiyeon Hong, Ji-Woong Park

**Affiliations:** School of Materials Science and Engineering and Research Institute for Solar and Sustainable Energies (RISE), Gwangju Institute of Science and Technology (GIST), 123, Cheomdan-gwagiro, Buk-gu, Gwangju 61005, Korea; chingukim@gist.ac.kr (C.K.); hjy8327@gist.ac.kr (J.H.)

**Keywords:** conducting polymers, selenium nanoparticles, thermoelectric, composites

## Abstract

We synthesized a hybrid nanocomposite comprised of selenium nanoparticles coated with a thin layer of a conductive polymer, poly(3,4-ethylenedioxythiophene), and studied its thermoelectric properties. The conductive polymer layer on the surface of nanoparticles in the composites formed a percolating network running between the stacked nanoparticles, exhibiting an electrical conductivity close to or higher than that of pure polymer. The thermoelectric power factor of the resulting composite was higher than that of individual polymer or selenium nanoparticles. We further increased the electrical conductivity of the composite by thermal annealing, thereby improving the power factor to 15 μW/cmK^2^ which is nine times higher than that of the polymer.

## 1. Introduction

Conductive polymers have been intensively studied in the past decade as an alternative thermoelectric material owing to their low thermal conductivity and low material cost as compared with inorganic thermoelectric materials. The thermoelectric efficiency, determined by figure of merit ZT = S^2^σT/κ (where S is the Seebeck coefficient, σ is electrical conductivity, T is temperature, and κ is thermal conductivity) of the conductive polymer has been significantly improved by optimization of doping level [1,2], post-processing [3,4,5], and hybridization with inorganic materials [6,7,8]. 

Hybridization of different materials at the nanoscale often allows us to take advantage of the strength of each constituent, and thus improvement of thermoelectric properties may be achieved by combining the conductive organic polymer with inorganic material [9,10,11]. Among various inorganic thermoelectric materials, selenium is a promising candidate for hybridization with conductive polymers. Selenium (Se) has a high Seebeck coefficient at room temperature, exceeding 1000 μV/K [12,13], and can be easily synthesized in nanocrystal form which could be transformed to different thermoelectric materials such as lead selenide (PbSe) and silver selenide (Ag_2_Se) by chemical transformation [14,15,16,17]. The electrical conductivity of Se crystal, however, is very low (in the range of 1 × 10^−5^–1 × 10^−6^ S/cm [13,18]); thus, the thermoelectric performance of Se itself is too poor to be useful for any application.

To increase the electrical conductivity of the Se-based composite, conductive polymer can be used as a conducting filler, which must be percolated in the composite even in a small composition to take advantage of the high Seebeck coefficient of Se. Coating the conductive polymers on the surface of colloidal particles is an attractive strategy to reduce the amount of the conductive polymer fillers by forming segregated conducting networks across the stacked particles [19,20]. 

**Figure 1:**
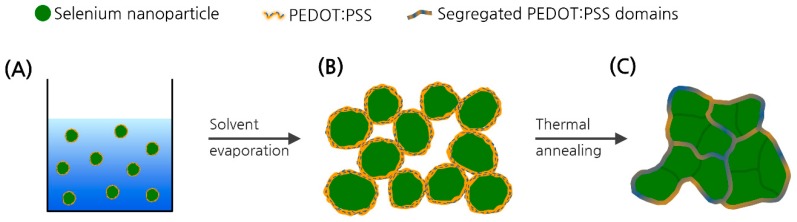
Schematic diagram of effective percolation of poly(3,4-ethylenedioxythiophene):poly(styrene sulfonate) (PEDOT:PSS) between stacked Se nanoparticles in the PEDOT:PSS/Se composite film. (**A**) PEDOT:PSS/Se core-shell nanoparticles in solution. (**B**) Segregated networks of PEDOT:PSS between the stacked particles after solvent evaporation. (**C**) Further segregated polymer networks from the sintered particles upon thermal annealing to form more continuous domains running across the Se domains.

Herein, we synthesized a hybrid nanocomposite material consisting of Se nanoparticles and a conductive polymer, poly(3,4-ethylenedioxythiophene):poly(styrene sulfonate) (PEDOT:PSS). The Se nanoparticles were prepared by the reduction of sodium selenite in the presence of PEDOT:PSS in aqueous solution. A thin layer of PEDOT:PSS with the thickness of a few nanometers formed on the surface of Se nanoparticles, providing a stable dispersion of PEDOT:PSS/Se core-shell nanoparticles. The film prepared by casting the PEDOT:PSS/Se solution showed an electrical conductivity of 0.37 S/cm with only approximately 4 wt % of polymer, which was almost the same as that of pristine PEDOT:PSS films and increased when compared to that of pristine Se by a factor of 1 × 10^6^, indicating that the polymer was effectively percolated between the stacked Se nanoparticles (Figure 1). Although the conductive polymer/Se nanoparticles exhibit a relatively low thermoelectric power factor (S^2^σ) of about 15 μW/cmK^2^, our approach demonstrates the promising potential of conductive polymer/Se nanocomposites for developing new thermoelectric materials.

## 2. Results and Discussion

**Figure 2:**
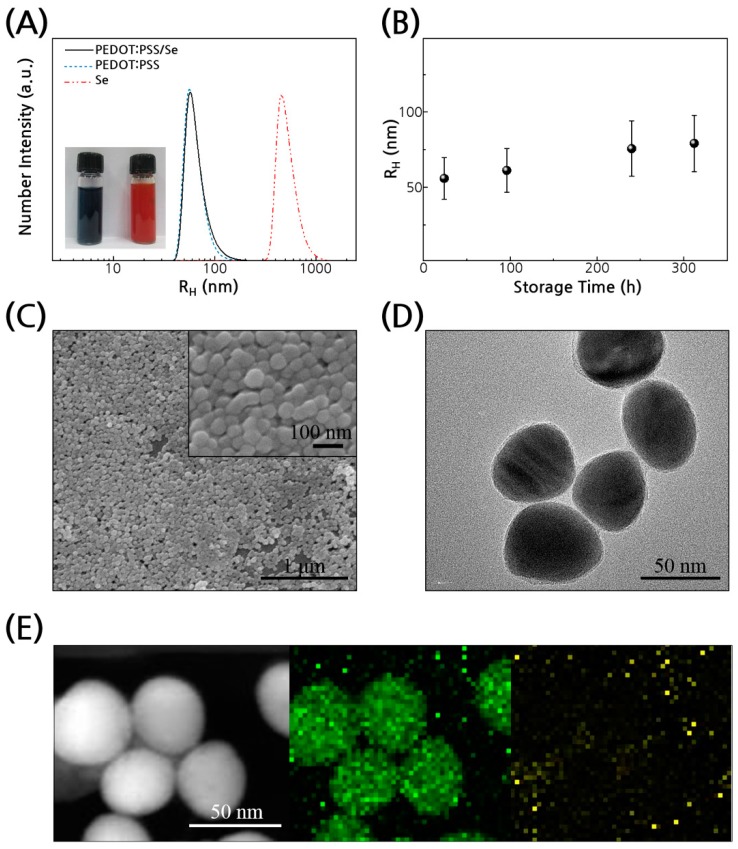
Structure of PEDOT:PSS/Se nanoparticles. (**A**) Dynamic light scattering (DLS) traces of PEDOT:PSS/Se, PSS/Se, and Se particles. a.u., arbitrary unit. Inset: photograph of aqueous solution of PEDOT:PSS (left) and PEDOT:PSS/Se nanoparticles (right). (**B**) The hydrodynamic radius (R_H_) of PEDOT:PSS/Se particles monitored for two weeks. (**C**) Scanning electron microscopy (SEM) and (**D**) Transmission electron microscopy (TEM) images of the composite particles. (**E**) High-angle annular dark-field scanning TEM (HAADF-STEM) image (left) and energy dispersive X-ray spectroscopy (EDS) mapping of Se (middle) and sulfur (right).

PEDOT:PSS/Se nanoparticles were synthesized by reducing sodium selenite with ascorbic acid in a dilute aqueous solution of PEDOT:PSS (2.7 × 10^−2^ wt %) at room temperature. Ascorbic acid reduces Se^4+^ to Se^0^ while PEDOT:PSS acts as a stabilizing agent of the nanoparticles. The reductant was dissolved in the PEDOT:PSS solution to which sodium selenite was added. Excess PEDOT:PSS chains free from Se particles could be removed by multiple centrifugations to obtain only PEDOT:PSS/Se as solid sediment. A red colored solution was obtained by re-dispersing the solid in water (Figure 2A inset); the color was indicative of the formation of amorphous Se nanoparticles [21]. The average hydrodynamic radius (R_H_) values of the resulting PEDOT:PSS/Se particles were measured to be 63 nm by dynamic light scattering (DLS) (Figure 2A).

Addition of sodium selenite to the solution containing ascorbic acid but no PEDOT:PSS resulted in the formation of larger aggregates of Se (Figure S1), of which R_H_ was 540 nm. Another control experiment was carried out where sodium selenite was added to the solution of ascorbic acid and PSS mixture (no PEDOT) and gave well-dispersed Se nanoparticles without the formation of aggregates. The size of the PSS/Se particles was very close to that of the PEDOT:PSS/Se nanoparticles, indicating that particle dispersion was mainly aided by PSS. The zeta potentials of both the PEDOT:PSS/Se solution and the PSS/Se solution had higher negative values (−38.9 and −41.6, respectively) than that of the Se particles without PSS (−26.9), suggesting the presence of negatively charged PSS chains on the Se nanoparticle surfaces (Figure S2).

The aqueous solution of PEDOT:PSS/Se nanoparticles was stable for a long time without agglomeration and sedimentation as shown in Figure 2B, which was monitored by the DLS-measured R_H_ for 2 weeks. Scanning and transmission electron microscopy (SEM and TEM) images of the particles showed that spherical Se nanoparticles were coated with a uniform ultrathin layer (2–3 nm) (Figure 2C,D). Energy dispersive X-ray spectroscopy (EDS) elemental mapping of Se and sulfur confirmed that the nanoparticle core consists of Se, and that its surface was covered with the PEDOT:PSS layer (Figure 2E). 

The weight fraction of PEDOT:PSS in the dry PEDOT:PSS/Se composite appeared to be about 4 wt % as determined by elemental analysis (Table S1). The R_H_ and SEM images of the composite particles synthesized in the different concentrations of PEDOT:PSS solution were shown in Figures S3 and S4, respectively. The particle size and shape changed negligibly when the concentration of PEDOT:PSS was higher than a critical value below which Se particles of larger sizes formed due to an insufficient amount of stabilizer.

Thin films of PEDOT:PSS/Se nanoparticles were prepared by drop-casting the composite solution on a slide glass (Figure S5). Thermoelectric properties of the composite films and each of the constituents are compared as shown in Table 1. The electrical conductivity and Seebeck coefficient of the PEDOT:PSS films exhibited similar values to those reported previously [6,10,22]. A pellet prepared from Se nanoparticles showed electrical conductivity in the order of 1 × 10^−7^ S/cm lower than that of bulk crystals. The Seebeck coefficient of the Se pellet could not be measured on our measurement set-up (Figure S6) because the signals of Seebeck voltage were hardly obtained owing to its too-high electrical resistance.

**Figure 3:**
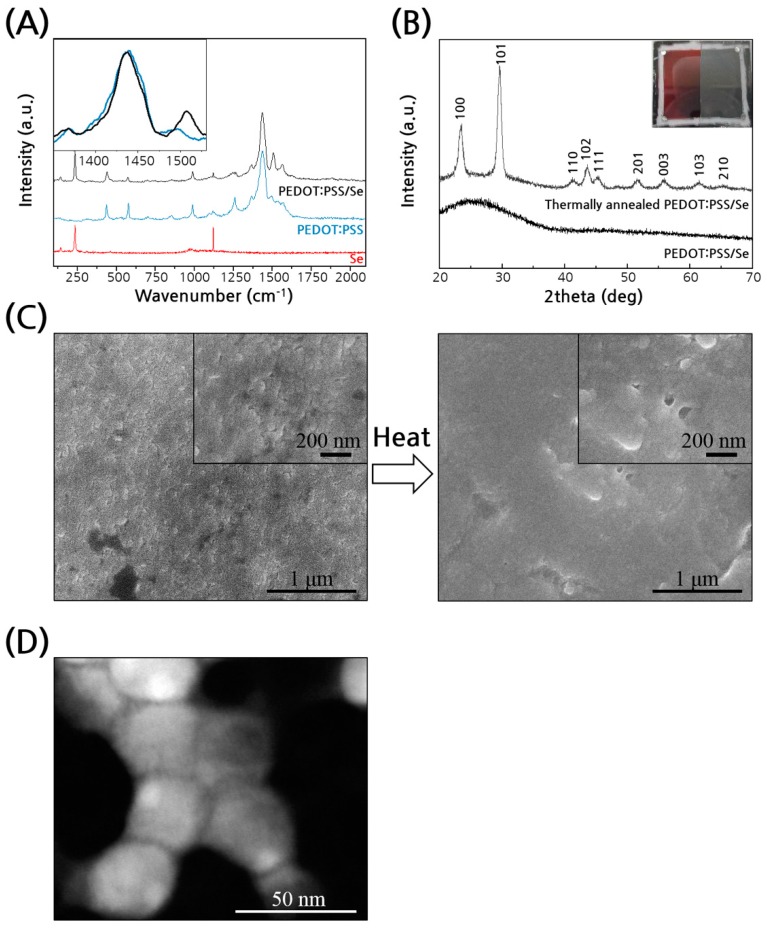
(**A**) Raman scattering spectra of PEDOT:PSS/Se, PEDOT:PSS, and Se. Effects of thermal annealing of the composite films. (**B**) X-ray diffraction (XRD) traces. Inset: photograph of the composite films before (left) and after of thermal annealing (right). (**C**) SEM images of top surfaces of the composite films before (left) and after thermal annealing (right). (**D**) HAADF-STEM image of thermally annealed composites.

Although the composite had only 4% of the conductive polymer, its electrical conductivity was nearly the same as that of the polymer itself, indicating the PEDOT:PSS domains in the composite film were effectively percolated between stacked Se particles. Additionally, Raman spectra indicated an expanded conformation of the PEDOT chain on the particle surfaces as shown in Figure 3A, where the symmetric C_α_ = C_β_ stretching peak is narrower and shifted to a low wavenumber as compared with the peak of pristine PEDOT:PSS, showing that the quinoid structures of PEDOT developed well in the PEDOT:PSS/Se composite [23,24]. This result indicates that charge transport through PEDOT was not hindered at the highly curved interfaces between the Se nanoparticles. Furthermore, the conductivity of PEDOT:PSS/Se could be increased further while maintaining the Seebeck coefficient by a brief thermal annealing of the films at 120 °C. As a result, the power factor of the composite could be increased to 15 μW/cmK^2^, which is nine times that of the polymer itself. The thermoelectric properties of the composite films annealed at different temperatures are shown in Table S2. The electrical conductivity was improved when the annealing temperature increased to 120 °C, and it was slightly decreased at 150 °C while maintaining the Seebeck coefficient. At 200 °C, both the conductivity and Seebeck coefficient decreased, most likely due to thermal degradation of the polymer.

We looked at how the thermal treatment affected the structure of the composite films by X-ray diffraction (XRD), SEM and TEM analysis. The amorphous Se in PEDOT:PSS/Se nanoparticles transformed into the crystalline trigonal phase upon annealing (Figure 3B) [25,26]. The color of the composite films changed from red to grey which is the typical color of Se in crystalline form (Figure 3B inset). The SEM images show that the morphology changed to more smooth and continuous structures with annealing than that of the as-prepared composite samples (Figure 3C) and the TEM revealed that some Se particles fused together (Figure 3D). The thickness of the composite films was also reduced to about 60 % of the as-prepared film after annealing. These data suggest that thermal annealing caused inter-particle sintering and crystallization of Se nanoparticles. However, it is unlikely that the electrical conductivity increase was a direct result of the crystallization of amorphous Se particles because both amorphous (≤1 × 10^−11^ S/cm) [27] and crystalline (1 × 10^−7^ S/cm) Se are poorly conductive compared with PEDOT:PSS. We postulate that the PEDOT chains were segregated further from the Se particles upon thermal annealing and reorganized to form domains of higher connectivity. It has been shown that the conductivity of PEDOT:PSS thin film increases upon thermal annealing [28]. Heating the PEDOT:PSS film removes its adsorbed water, enhancing segregation of the polymer chains and interconnectivity of the PEDOT domains [29,30]. It is reasonable to assume that the morphological change occurs similarly to the PEDOT:PSS in our composite films. 

**Figure 4:**
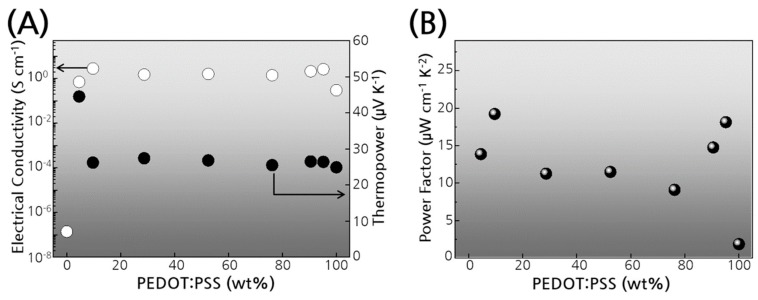
Thermoelectric properties of PEDOT:PSS/Se composite films by varying the composition between PEDOT:PSS and Se. (**A**) Electrical conductivity and Seebeck coefficient. (**B**) Power factor.

The effect of the relative compositions of PEDOT:PSS and Se on the thermoelectric properties was studied with the samples prepared by adding pure PEDOT:PSS solution to the original composite solution (Figure 4). The electrical conductivity increased to 2.8 S/cm when the total amount of PEDOT:PSS was increased to about 10%, but no further increase was achieved with more polymer (Figure 4A). It is likely that the extra polymer filled the voids or disconnected gaps between the particles and thus improved the bulk conductivity (Figure S7). Many conductive porous materials have been shown to have an opposite dependency of electrical conductivity on its porosity [31,32,33] because porous material has a lower sectional area for electrical current than non-porous material. In contrast to the increase in electrical conductivity by addition of more polymer to the composite, the Seebeck coefficient of the composite decreased to the value of pristine PEDOT:PSS (Figure 4A). Nevertheless, the higher electrical conductivity of the hybrid nanocomposites resulted in improved power factors for all compositions as compared with pure polymer or Se (Figure 4B).

## 3. Conclusions

We synthesized PEDOT:PSS/Se core-shell nanoparticles as a new thermoelectric composite material. The Se particles were coated and stabilized with PEDOT:PSS in water and the solution was cast into thermoelectric composite films. The conductive PEDOT:PSS domains were percolated effectively even in a small composition, resulting in enhanced electrical conductivity when compared with individual components of the composites. The thermoelectric power factor of the composite was further increased by thermal annealing and optimization of the polymer composition.

## 4. Materials and Methods 

### 4.1. Materials

PEDOT:PSS aqueous solution (1.1 wt %, Clevios PH 1000, Heraeus, Hanau, Germany), sodium selenium (Na_2_SeO_3_) (99%, Aldrich, Yongin, Korea), ascorbic acid (>99%, Aldrich, Yongin, Korea), and polystyrene sulfonate (PSS) (75 k, aqueous solution of 18 wt %, Aldrich, Yongin, Korea) were used as received. The thermoelectric standard reference sample of bismuth telluride (Bi_2_Te_3_) was purchased from NIST (SRM 3451, Gaithersburg, MD, USA).

### 4.2. Synthesis of PEDOT:PSS/Se, PSS/Se, Se Particles Aqueous Solution

For the synthesis of the PEDOT:PSS/Se nanoparticle solution, the commercial PEDOT:PSS solution (1.1 wt % in water, 2 mL) and ascorbic acid (2 g, 11.4 mmol) were dissolved in water (80 mL). The synthesis of Se particles was started by adding sodium selenite powder (0.104 g, 0.60 mmol) into the solution with magnetic stirring. The navy black solution immediately appeared reddish when Na_2_SeO_3_ was added. After 5 h of reaction at room temperature, the solution was centrifuged at 15,000 rpm for 25 min. The supernatant was carefully discarded and the brick-red sediments were resuspended in double-distilled water by vigorous vortexing. This cycle was repeated three times to remove the side products and the excess amount of PEDOT:PSS. The PEDOT:PSS/Se nanoparticle solution was finally obtained by dispersing the sediments in 4 ml water. The resulting aqueous dispersion had an average concentration of 0.53 wt %.

For the PSS/Se synthesis, the as-purchased PSS solution was diluted with water so that 2.7 × 10^−2^ wt % PSS in 80 mL aqueous solution was obtained, to which ascorbic acid (2 g, 11.4 mmol) was dissolved. The synthesis of Se particles in the resultant PSS solution was the same as that of PEDOT:PSS/Se. 

The Se particles were prepared following the same process, but in the absence of dispersants such as PEDOT:PSS or PSS. The resulting Se powders were dried at 80 °C for 24 h in vacuo.

### 4.3. Fabrication of the Films (or Pellets)

#### 4.3.1. PEDOT:PSS/Se Films

The PEDOT:PSS/Se aqueous solution was drop-casted on oxygen plasma treated slide glass and dried at room temperature in air, in the dark for 24 h (PEDOT:PSS/Se film), followed by thermal annealing on a hotplate (120 °C) for 15 min in air (thermally annealed film). 

#### 4.3.2. PEDOT:PSS Films

The PEDOT:PSS films were fabricated by drop-casting the PH1000 solution on the oxygen plasma treated slide glass and dried at room temperature in air for 24 h, followed by thermal annealing on a hotplate (120 °C) for 15 min in air.

#### 4.3.3. Se Pellets

The Se pellet was prepared by compressing of finely ground Se powder.

### 4.4. Characterization

The hydrodynamic radius and zeta potential of the nanoparticles were estimated by electrophoretic light scattering (ELS-Z, Otsuka Electronics Co., Osaka, Japan). The sample solutions were diluted by a factor of 1000 and then filtered (pore size 0.45 μm, cellulose acetate, ADVANTEC, Tokyo, Japan) just before the measurement, and the Se particle dispersed solution was not filtered for the measurement. The samples were measured at least three times and the values were averaged. The amount of polymer in the composite was estimated with elemental analysis (EA2000 and EA2111, Thermo Finnigan, San Jose, CA, USA). The morphology of particles was analyzed with SEM (JSM-6700F, JEOL Korea LTD, Seoul, Korea) and TEM (G2 F30 S-twin, Tecnai, Hillsboro, OR, USA) equipped with an EDS system (EDAX, AMETEK, Mahwah, NJ, USA). Raman spectra (inVia Raman Microscope, Renishaw, Wotton-under-Edge, UK) was obtained with 514 nm excitation wavelength from an Ar^+^ laser source (0.2 mW laser power). The crystal structures of the films were studied with an X-ray diffractometer with 100 mA at 40 kV (Rint 2000, Rigaku, Tokyo, Japan). 

### 4.5. Thermoelectric Property

Electrical conductivity was measured by Hall measurement with van der Pauw configuration (HL5500PC, Bio-Rad, Hercules, CA, USA). For better electrical contact, silver paste was used as electrodes on the specimens. Because the periphery of the drop-cast film was thicker than the film center, we cut off the film edge (about 30–40% of the as-coated film area) to make more uniform films before measuring the resistance as shown in the inset of Figure 3B. In addition, the thickness of each film was averaged over four different points of the film including both central and peripheral regions. The standard deviations of thickness of ten samples were 3–13%. The Seebeck coefficient of thin films (a few μm thick) was measured by a homemade set-up in air as shown in Figure S6. Seebeck voltage and temperature were measured by a digital multimeter (Keithley 2700). Two K-type thermocouples were located on each side of temperature control blocks. The full span of temperature difference was 4–5 K across the films. As shown in Figure S8, the measurement was calibrated with a standard reference sample (Bi_2_Te_3_ bar). The error range was about 4%, which might be mainly from the thermal contact resistance between the specimen and thermocouples. The thermoelectric properties of PEDOT:PSS/Se films with different thicknesses from 1.4–4.5 μm were compared as shown in Table S3.

## Figures and Tables

**Table 1:** Thermoelectric properties of PEDOT:PSS/Se and control films at room temperature.

System	Electrical Conductivity (σ) / S cm^−1^	Seebeck Coefficient (S) / μV K^−1^	Power Factor (S^2^σ) / μW cm^−1^K^−2^
**Se ^a^**	1.4 x 10^−7^	~1000 ^b^	~0.0014
**PEDOT:PSS**	0.29 (±0.17)	24.9 (±0.9)	1.7
**PEDOT:PSS/Se**	0.37 (±0.19)	45.5 (±3.5)	9.5
**PEDOT:PSS/Se ^c^**	0.71 (±0.10)	44.5 (±6.7)	15.0

^a^ The Se pellet was prepared by compression of finely ground Se powders. ^b^ Bulk Seebeck coefficient of Se crystals [12,13]. ^c^ Thermally annealed films of PEDOT:PSS/Se at 120 °C for 15 min.

## Supplementary Materials

The following are available online at https://www.mdpi.com/2073-4360/11/6/1052/s1.
