# Supplementary material for: Synthesis and Thermoelectric Properties of Selenium Nanoparticles Coated with PEDOT:PSS
Source: Polymers (Basel). 2019 Jun 17;11(6):1052. doi: 10.3390/polym11061052 (PMC6631964; doi:10.3390/polym11061052)
Supplement: Supplementary file 1 [file polymers-11-01052-s001.pdf]

Supporting Information

Synthesis and Thermoelectric Properties of Selenium Nanoparticles coated with PEDOT:PSS

Chingu Kim, Jiyeon Hong, and Ji-Woong Park *

School of Materials Science and Engineering and Research Institute for Solar and Sustainable Energies (RISE), Gwangju Institute of Science and Technology (GIST), 123, Cheomdan-gwagiro, Buk-gu, Gwangju 61005, Korea.

E-mail: jiwoong@gist.ac.kr

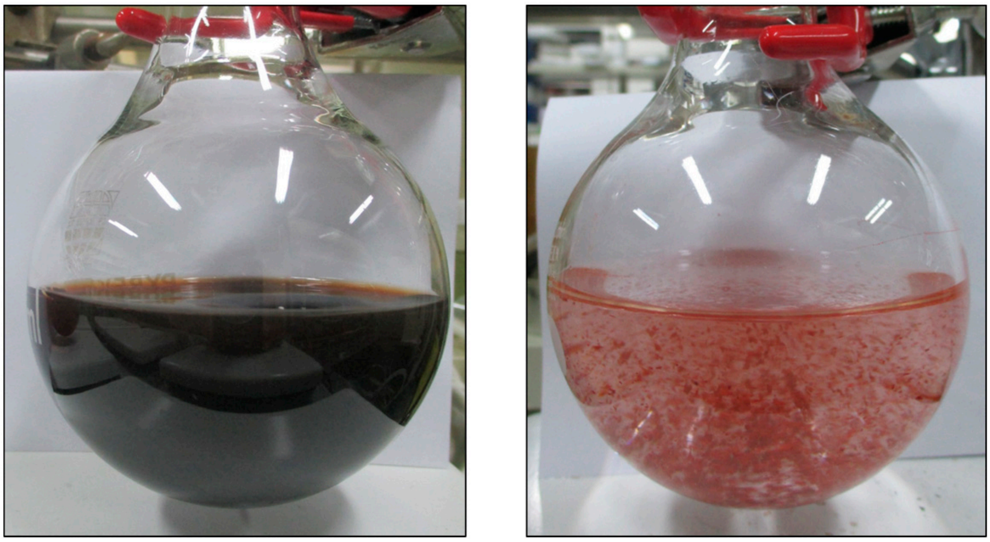

Fig. S1. Photographs of reaction mixtures with or without PEDOT:PSS (left and right, respectively).

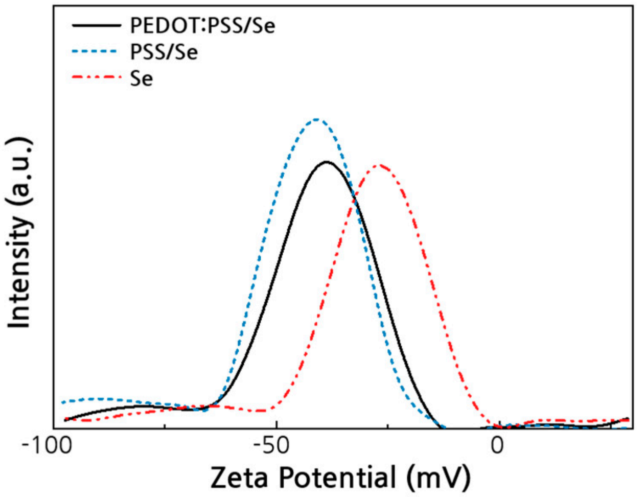

Fig. S2. Zeta potential distributions of PEDOT:PSS/Se, PSS/Se, Se particles dispersed aqueous solutions.

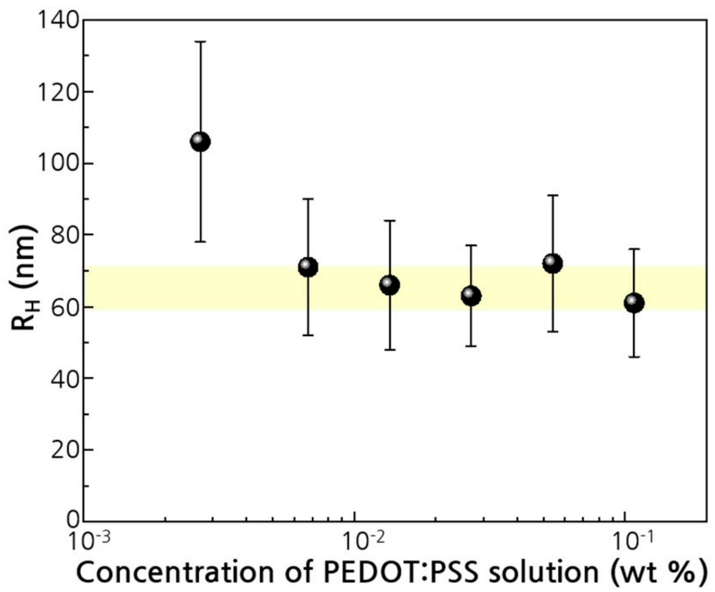

Fig. S3. Hydrodynamic radius of PEDOT:PSS/Se particles synthesized with different concentration of PEDOT:PSS solution. The yellow shade indicates the R_H from 60 to 70 nm.

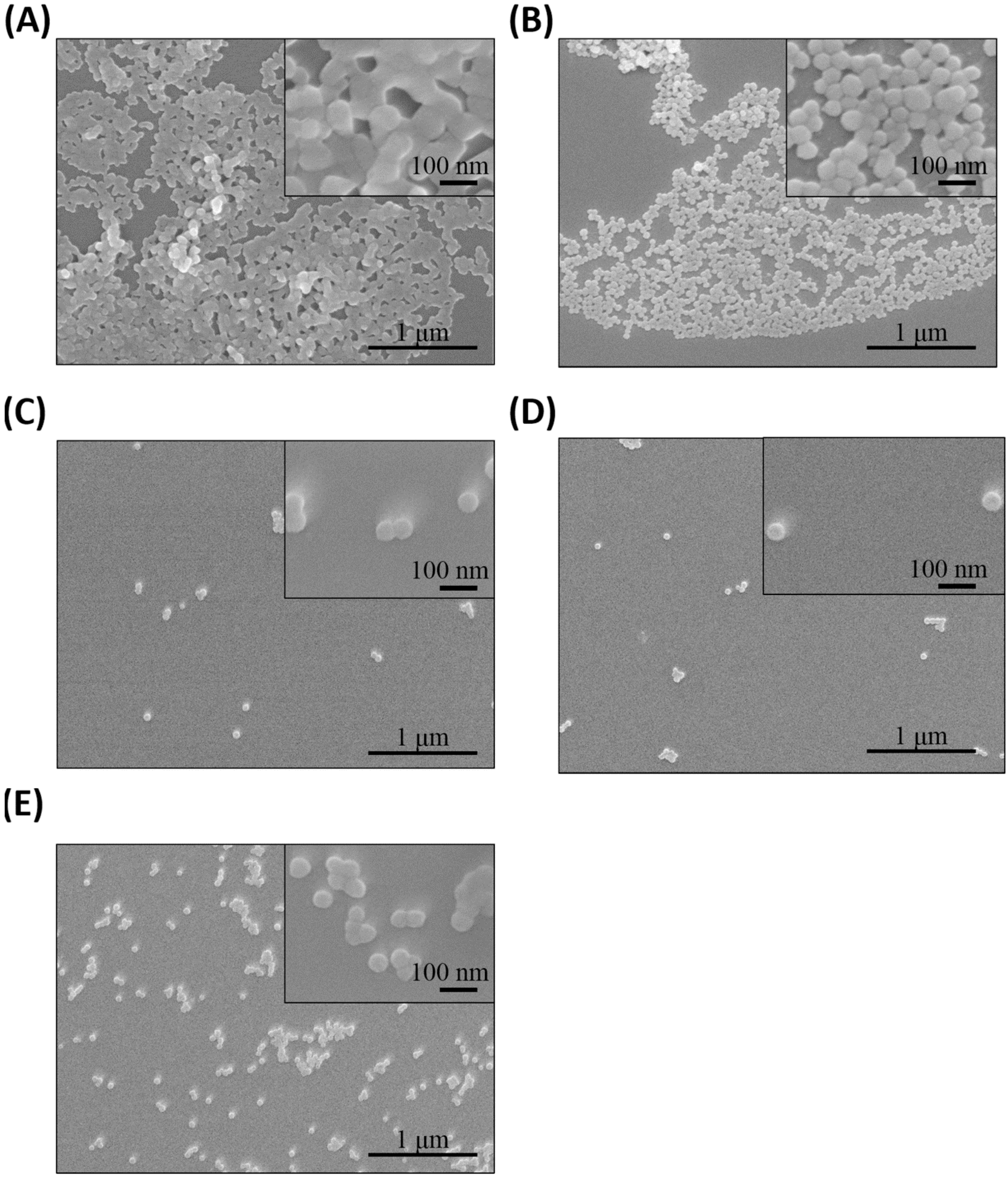

Fig. S4. SEM images of the PEDOT:PSS/Se particles synthesized with different concentration of PEDOT:PSS solution. (A) 2.7 x 10^{-3} %. (B) 6.75 x 10^{-3} %. (B) 1.35 x 10^{-2} %. (B) 5.4 x 10^{-2} %. (B) 1.08 x 10^{-1} %.

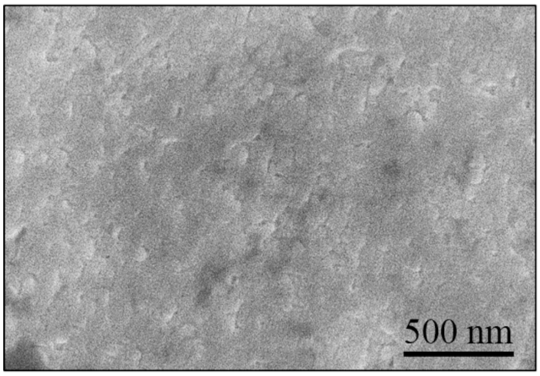

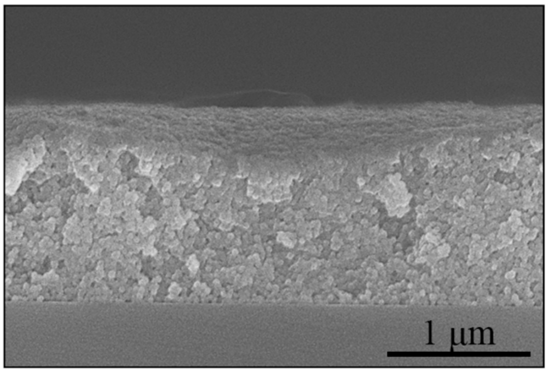

Fig. S5. SEM images of top surface (left) and cross-section (right) of PEDOT:PSS/Se film.

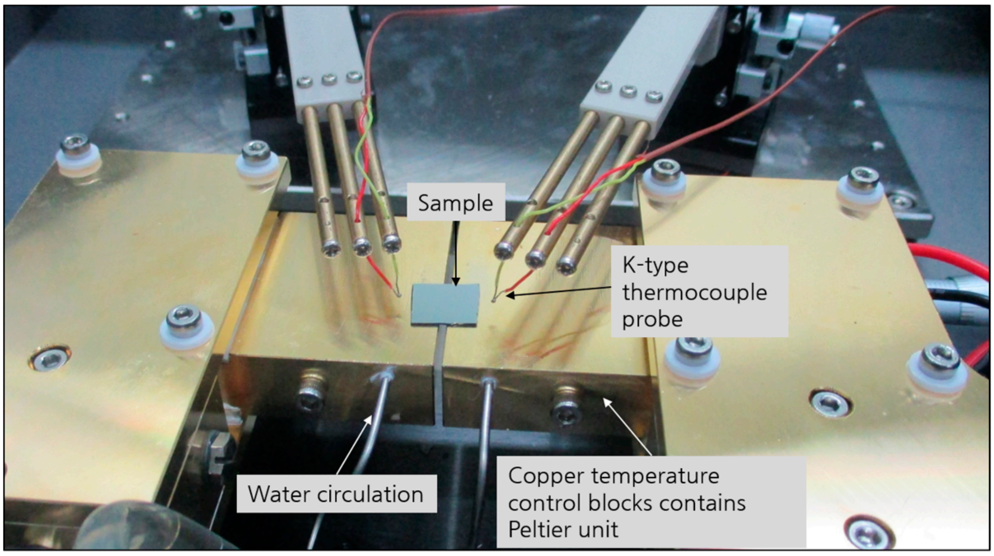

Fig. S6. Seebeck measurement set-up. Seebeck voltage was measured at different temperatures.

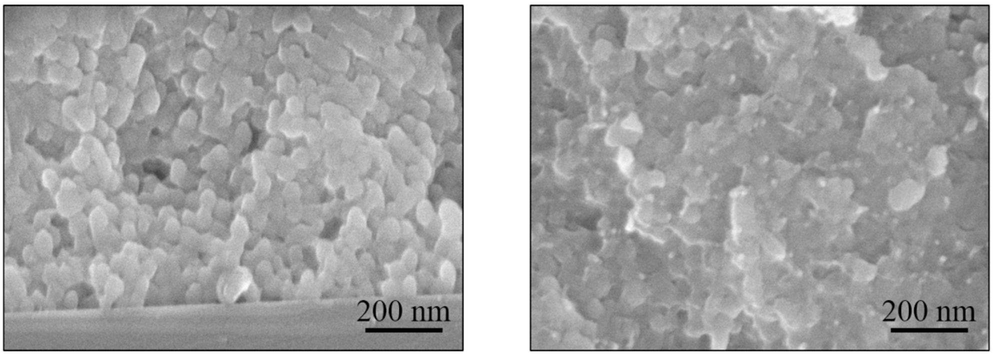

Fig. S7. SEM images of cross-sections of the composites films of about 4% (left) and 10% polymer compositions (right).

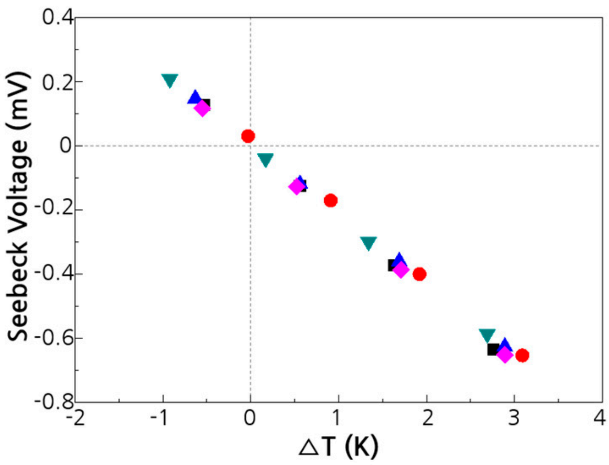

Fig. S8. Measurement of Seebeck voltage at different temperatures of a standard sample (Bi_2Te_3 bar) five times.

Table S1. Polymer content estimated from elemental analysis of PEDOT:PSS/Se composite.

	C	H	O	S	Total
Measured (%)	1.99	-	-	0.87	
Estimated [a] (on C basis, %)	1.99	0.15	0.96	0.73	3.8
(on S basis, %)	2.39	0.18	1.16	0.87	4.6

[a] The calculation was made based on following assumptions. 1) The weight ratio of PEDOT/PSS (Clevios PH 1000, Heraeus) is 1/2.5. 2) Degree of sulfonation of PSS is 100 %.

Table S2. Room temperature thermoelectric properties of PEDOT:PSS/Se films which were thermally annealed at different temperatures.

**Annealing Temperature (T) / °C**	**Electrical Conductivity (σ) / S cm^{-1}**	**Seebeck Coefficient (S) / μV K^{-1}**	**Power Factor ($S^2\sigma$) / μW cm^{-1}K^{-2}**
No	0.37	45.5	9.5
70	0.27	43.4	5.1
100	0.37	43.5	7.0
120	0.71	44.5	15.0
150	0.49	45.2	10.0
200	0.14	40.7	2.3

Table S3. Room temperature thermoelectric properties of PEDOT:PSS/Se films with different thicknesses.

**Average film thickness / μm**	**Electrical Conductivity (σ) / S cm^{-1}**	**Seebeck Coefficient (S) / μV K^{-1}**	**Power Factor ($S^2\sigma$) / μW cm^{-1}K^{-2}**
1.4	0.58	43.2	10.8
3.0	0.69	45.0	14.0
4.5	0.57	44.9	11.5
